# Draft Genome Sequence of the First NDM-4-Producing *Escherichia coli* Strain (AK1), Isolated from Sewage Water of a North Indian Hospital

**DOI:** 10.1128/genomeA.01366-17

**Published:** 2017-12-14

**Authors:** Asad U. Khan, Ayesha Z. Beg, Praveen K. Verma

**Affiliations:** a Medical Microbiology and Molecular Biology Lab, Interdisciplinary Biotechnology Unit, AMU, Aligarh, India; b National Institute of Plant Genome Research, New Delhi, India

## Abstract

We report here the draft genome sequence of the first isolated NDM-4-producing *Escherichia coli* strain, isolated from sewage water at a North Indian hospital. The genome has an assembly size of 5,076,053 bp, arranged in 129 contigs, with 5,271 genes and a G+C content of 50.47%.

## GENOME ANNOUNCEMENT

The escalated spread of antibiotic-resistant bacteria is an alarming situation in the context of treating infection (1). Carbapenem-resistant *Enterobacteriaceae* strains contain an enzyme, carbapenemase, that hydrolyzes β-lactams of all classes (2). The enterobacterium *Escherichia coli* possesses the carbapenemase New Delhi metallo-β-lactamase (NDM), which is commonly associated with multidrug resistance genes, endowing a very high level of drug resistance and rendering it unassailable (3, 4). Here, we present the genome sequence of the NDM-4-carrying *E. coli* strain AK1 to understand the genes associated with its resistance and virulence and gain further information about its pathogenicity.

The *E. coli* isolate was collected from hospital sewage in 2011 (5). The bacterial DNA was isolated using a Qiagen QIAamp DNA minikit, and GE SimpliNano, a UV-visible spectrophotometer, was used to measure the concentration and purity of the DNA. The DNA was subjected to whole-genome sequencing on an Illumina NextSeq 500 platform using the 2 × 150-bp paired-end read length sequencing protocol. The raw sequence data were further analyzed by FastQ for quality control purposes. SPAdes version 3.10.1 was used to create a *de novo* assembly with a genome coverage of 266.181×. Genome annotation was performed with the NCBI Prokaryotic Genome Annotation Pipeline using a best-placed reference protein set and GeneMarkS+ methodology. The genome was further analyzed with VirulenceFinder (6), CARD/ARDB (7), BLASTn searches for O and H alleles, and plasmid multilocus sequence typing (http://www.pubmlst.org/plasmid) (8) for virulence factors, resistance genes, serotype, incompatibility group of plasmid, and sequence type (ST) of the isolate.

The genome analysis revealed that the isolate belongs to ST315 and has the serotype O15:H7. The genome assembly consists of 129 contigs and has a sequence length of 5,076,053 bp with an overall G+C content of 50.74%. The genome was identified to have 5,271 genes, out of which 4,948 are coding sequences for proteins. Due to the unavailability of a genome sequence for *E. coli* ST315, a BLAST analysis of this genome was done, and the subject genome with highest identity, *Escherichia coli* O157:H7 strain Sakai (GenBank accession no. NC_002695), was used as the reference. The analysis of the genome revealed the presence of virulence factors associated with diarrheagenic *E. coli* and a very high number of resistance genes (some control two-component systems). Included in the genome is a plasmid that is 155,678 bp long with a G+C content of 52% and belongs to the incompatibility group IncF. It harbors *bla*_NDM-4_, *bla*_CTX-M-15_, and *bla*_TEM-1b_ resistance genes for aminoglycosides and sulfonamide in association with class I integrons, transposons, and insertion sequence elements. Of note, *E. coli* ST315 belongs to human host and phylogenetic group D (9).

### Accession number(s).

This whole-genome shotgun project (PRJNA397500) of *E. coli* strain AK1 has been deposited at DDBJ/ENA/GenBank under the accession no. NSBV00000000.
